# 2,5-Bis[2-(2-methoxy­ethoxy)phen­yl]-1,3,4-oxadiazole

**DOI:** 10.1107/S1600536809028931

**Published:** 2009-07-29

**Authors:** Xia Tian, Xiao-Li Zhen, Jian-Rong Han, Chang-Xin Ming, Shou-Xin Liu

**Affiliations:** a College of Sciences, Hebei University of Science & Technology, Shijiazhuang 050018, People’s Republic of China; b College of Chemical & Pharmaceutical Engineering, Hebei University of Science & Technology, Shijiazhuang 050018, People’s Republic of China

## Abstract

In the title compound, C_20_H_22_N_2_O_5_, the central 1,3,4-oxadiazole ring is essentially planar [r.m.s. deviation from the best plane of 0.0011 Å] and makes dihedral angles of 4.10 (3) and 13.32 (4)° with the two benzene rings. In the crystal structure, the packing is stabilized by weak non-classical inter­molecular C—H⋯N hydrogen bonds, which link the mol­ecules into an extended network.

## Related literature

For the optical and electronic properties of 1,3,4-oxadizole and its dericatives, see: Emi & Toru (2006 ▶). Liu *et al.* (1997 ▶); Peng *et al.* (2006 ▶); Satoshi *et al.* (2000 ▶). For reference geometrical data: see: Tian *et al.* (2009 ▶). For related structures, see: Orgzall *et al.* (2005 ▶).
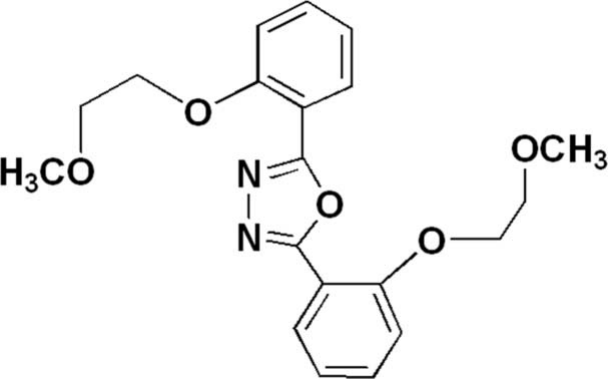


## Experimental

### 

#### Crystal data

C_20_H_22_N_2_O_5_
*M* *_r_* = 370.40
Monoclinic, 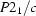

*a* = 7.7264 (15) Å
*b* = 13.886 (3) Å
*c* = 16.911 (3) Å
β = 96.42 (3)°
*V* = 1803.0 (6) Å^3^
*Z* = 4
Mo *K*α radiation
μ = 0.10 mm^−1^
*T* = 293 K
0.16 × 0.14 × 0.10 mm

#### Data collection

Rigaku Saturn diffractometer
Absorption correction: multi-scan (*CrystalClear*; Rigaku, 2005 ▶) *T*_min_ = 0.984, *T*_max_ = 0.990
13017 measured reflections
4290 independent reflections
3635 reflections with *I* > 2σ(*I*)
*R*_int_ = 0.030

#### Refinement

*R*[*F* ^2^ > 2σ(*F* ^2^)] = 0.040
*wR*(*F* ^2^) = 0.110
*S* = 1.04
4290 reflections
246 parameters
H-atom parameters constrained
Δρ_max_ = 0.25 e Å^−3^
Δρ_min_ = −0.23 e Å^−3^

### 

Data collection: *CrystalClear* (Rigaku, 2005 ▶); cell refinement: *CrystalClear*; data reduction: *CrystalClear*; program(s) used to solve structure: *SHELXS97* (Sheldrick, 2008 ▶); program(s) used to refine structure: *SHELXL97* (Sheldrick, 2008 ▶); molecular graphics: *SHELXTL* (Sheldrick, 2008 ▶); software used to prepare material for publication: *SHELXTL*.

## Supplementary Material

Crystal structure: contains datablocks I, global. DOI: 10.1107/S1600536809028931/pv2184sup1.cif

Structure factors: contains datablocks I. DOI: 10.1107/S1600536809028931/pv2184Isup2.hkl

Additional supplementary materials:  crystallographic information; 3D view; checkCIF report

## Figures and Tables

**Table 1:** Hydrogen-bond geometry (Å, °)

*D*—H⋯*A*	*D*—H	H⋯*A*	*D*⋯*A*	*D*—H⋯*A*
C5—H5⋯N2^i^	0.93	2.62	3.385 (2)	140

Symmetry code: (i) 
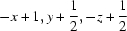
.

**Fig. 1.**
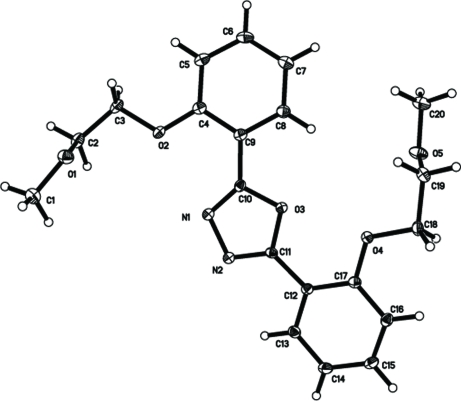
The molecular structure of (I) with displacement ellipsoids for non-H atoms drawn at the 30% probability level.

**Fig. 2.**
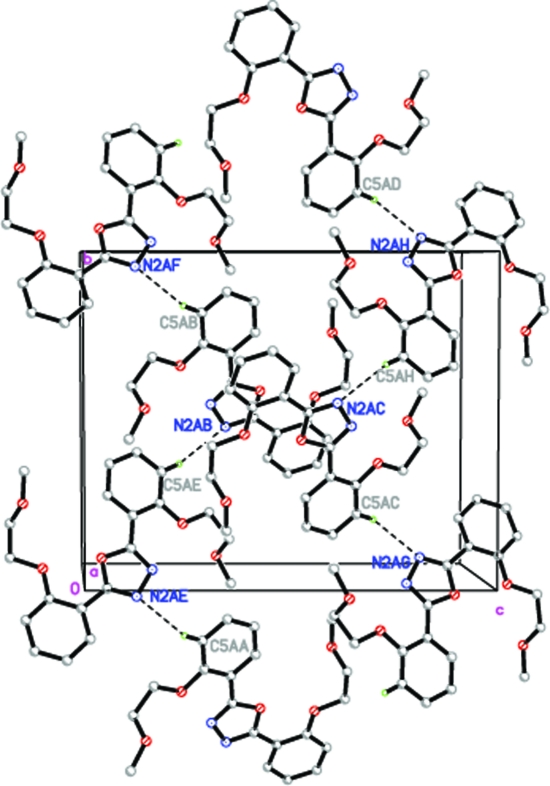
Packing diagram for (I), with H bonds drawn as dashed lines; H-atom not involved in interactions have been excludeed

## supplementary crystallographic information

### Comment

The optical and electronic properties of 1,3,4-oxadizole have received great attention in the field of electroluminescence (Emi *et al.*, 2006). A well known derivative of 1,3,4-oxadiazole, 2-(4-biphenyl)-5-(*tert*-butylphenyl)-1,3,4-oxadiazole (PBD), has been used as electron-injection material to improve the balance of charge carrier and to increase the photon/electron quantum efficiency (Liu *et al.*, 1997) and the electron-transporting material in organic electroluminescence device (Satoshi *et al.*, 2000). It has been demonstrated that modifying the side chains or inserting other heterocycles in 1,3,4-oxadizole system could result in good electroluminescent molecules as organic electroluminescence materials (Peng *et al.*, 2006). As part of an investigation on potential electroluminescent molecules by modifying the side chains of 2,5-diaryl-1,3,4-oxadizole, we reporte here the synthesis and structure of the title compound, (I).

The molecular structure of (I) is presented in Fig. 1. The oxadizole ring (O3/C10/N1/N2/C11) is essentially planar, with an r.m.s. deviation for fitted atoms of 0.0011 Å. It makes dihedral angles of, 13.32 (4) and 4.10 (3)°, respectively, with the benzene rings (C4—C9) and (C12—C17). The crystal packing is stabilized by weak non-classical intermolecular C—H···N hydrogen bonds which link the molecules into an infinite network. The bond lengths and angles in (I) are within their normal ranges (Tian *et al.* 2009). The crystal structure of a 2,5-diaryl-1,3,4-oxadiazole derivative have been reported (Orgzall *et al.*, 2005).

### Experimental

2,5-Di(*o*-hydroxyphenyl)-1,3,4-oxadiazole (0.8 g, 3.0 mmol), NaH (0.5 g, 20 mmol) and 1-chloro-2-methoxyethane (0.75 g, 8 mmol) were added and dissolved in 50 ml of THF, the mixture was stirred refluxing for 10 h. giving a colourless precipitate. The product was isolated, recrystallized from ethyl acetate then dried in a vacuum to give the pure compound in 81% yield. colourless single crystals of (I) suitable for X-ray analysis were obtained by slow evaporation of ethyl acetate solution at room temperature.

### Refinement

The H atoms were included in calculated positions (C—H = 0.93–0.97 Å) and refined as riding with *U*_iso_(H) = 1.2*U*_eq_(C) for aromatic and methylene H atoms and *U*_iso_(H) = 1.5*U*_eq_(methyl C).

### Crystal data

**Table:**

C_20_H_22_N_2_O_5_	*F*(000) = 784
*M**_r_* = 370.40	*D*_x_ = 1.364 Mg m^−^^3^
Monoclinic, *P*2_1_/*c*	Mo *K*α radiation, λ = 0.71073 Å
Hall symbol: -P 2ybc	Cell parameters from 4968 reflections
*a* = 7.7264 (15) Å	θ = 2.4–27.9°
*b* = 13.886 (3) Å	µ = 0.10 mm^−^^1^
*c* = 16.911 (3) Å	*T* = 293 K
β = 96.42 (3)°	Prism, colourless
*V* = 1803.0 (6) Å^3^	0.16 × 0.14 × 0.10 mm
*Z* = 4	

### Data collection

**Table:**

Rigaku Saturn diffractometer	4290 independent reflections
Radiation source: rotating anode	3635 reflections with *I* > 2σ(*I*)
confocal	*R*_int_ = 0.030
ω scans	θ_max_ = 27.9°, θ_min_ = 2.4°
Absorption correction: multi-scan (*CrystalClear*; Rigaku, 2005)	*h* = −9→10
*T*_min_ = 0.984, *T*_max_ = 0.990	*k* = −13→18
13017 measured reflections	*l* = −20→22

### Refinement

**Table:**

Refinement on *F*^2^	Primary atom site location: structure-invariant direct methods
Least-squares matrix: full	Secondary atom site location: difference Fourier map
*R*[*F*^2^ > 2σ(*F*^2^)] = 0.040	Hydrogen site location: inferred from neighbouring sites
*wR*(*F*^2^) = 0.110	H-atom parameters constrained
*S* = 1.04	*w* = 1/[σ^2^(*F*_o_^2^) + (0.0696*P*)^2^ + 0.0999*P*] where *P* = (*F*_o_^2^ + 2*F*_c_^2^)/3
4290 reflections	(Δ/σ)_max_ < 0.001
246 parameters	Δρ_max_ = 0.25 e Å^−^^3^
0 restraints	Δρ_min_ = −0.23 e Å^−^^3^

### Special details

**Table:**

Geometry. All e.s.d.'s (except the e.s.d. in the dihedral angle between two l.s. planes) are estimated using the full covariance matrix. The cell e.s.d.'s are taken into account individually in the estimation of e.s.d.'s in distances, angles and torsion angles; correlations between e.s.d.'s in cell parameters are only used when they are defined by crystal symmetry. An approximate (isotropic) treatment of cell e.s.d.'s is used for estimating e.s.d.'s involving l.s. planes.
Refinement. Refinement of *F*^2^ against ALL reflections. The weighted *R*-factor *wR* and goodness of fit *S* are based on *F*^2^, conventional *R*-factors *R* are based on *F*, with *F* set to zero for negative *F*^2^. The threshold expression of *F*^2^ > σ(*F*^2^) is used only for calculating *R*-factors(gt) *etc*. and is not relevant to the choice of reflections for refinement. *R*-factors based on *F*^2^ are statistically about twice as large as those based on *F*, and *R*- factors based on ALL data will be even larger.

### Fractional atomic coordinates and isotropic or equivalent isotropic displacement parameters (Å^2^)

**Table:**

	*x*	*y*	*z*	*U*_iso_*/*U*_eq_	
O1	0.88655 (10)	0.03469 (6)	0.35096 (5)	0.0284 (2)	
O2	0.63462 (10)	0.15549 (5)	0.25616 (5)	0.02362 (19)	
O3	0.35245 (9)	0.06943 (5)	0.04514 (4)	0.01871 (17)	
O4	0.23221 (10)	0.04689 (5)	−0.10258 (4)	0.02263 (18)	
O5	0.18138 (11)	0.24756 (6)	−0.15011 (6)	0.0307 (2)	
N1	0.40253 (12)	0.02963 (6)	0.17243 (6)	0.0230 (2)	
N2	0.30539 (12)	−0.04406 (6)	0.13138 (6)	0.0228 (2)	
C1	0.89595 (18)	−0.05579 (9)	0.38968 (8)	0.0337 (3)	
H1A	0.9211	−0.0464	0.4460	0.051*	
H1B	0.9865	−0.0937	0.3706	0.051*	
H1C	0.7866	−0.0886	0.3787	0.051*	
C2	0.76278 (15)	0.09686 (9)	0.38010 (7)	0.0265 (3)	
H2A	0.8027	0.1161	0.4342	0.032*	
H2B	0.6524	0.0636	0.3804	0.032*	
C3	0.73965 (15)	0.18388 (8)	0.32781 (7)	0.0245 (2)	
H3A	0.6825	0.2348	0.3543	0.029*	
H3B	0.8519	0.2073	0.3156	0.029*	
C4	0.60454 (14)	0.22178 (7)	0.19721 (7)	0.0207 (2)	
C5	0.66365 (15)	0.31641 (8)	0.20354 (7)	0.0251 (2)	
H5	0.7257	0.3378	0.2506	0.030*	
C6	0.62996 (15)	0.37886 (8)	0.13967 (8)	0.0281 (3)	
H6	0.6681	0.4423	0.1445	0.034*	
C7	0.53997 (16)	0.34777 (8)	0.06861 (8)	0.0280 (3)	
H7	0.5217	0.3894	0.0254	0.034*	
C8	0.47746 (15)	0.25413 (8)	0.06264 (7)	0.0235 (2)	
H8	0.4158	0.2335	0.0153	0.028*	
C9	0.50575 (14)	0.19043 (7)	0.12671 (6)	0.0197 (2)	
C10	0.42605 (13)	0.09472 (7)	0.11946 (6)	0.0182 (2)	
C11	0.27775 (13)	−0.01706 (7)	0.05765 (6)	0.0176 (2)	
C12	0.17690 (13)	−0.06928 (7)	−0.00691 (6)	0.0177 (2)	
C13	0.09329 (14)	−0.15339 (7)	0.01298 (7)	0.0219 (2)	
H13	0.1025	−0.1731	0.0658	0.026*	
C14	−0.00307 (15)	−0.20810 (8)	−0.04446 (7)	0.0250 (2)	
H14	−0.0577	−0.2642	−0.0304	0.030*	
C15	−0.01745 (15)	−0.17859 (8)	−0.12307 (7)	0.0245 (2)	
H15	−0.0796	−0.2161	−0.1620	0.029*	
C16	0.05963 (14)	−0.09390 (8)	−0.14445 (7)	0.0219 (2)	
H16	0.0471	−0.0741	−0.1973	0.026*	
C17	0.15615 (13)	−0.03817 (7)	−0.08668 (6)	0.0185 (2)	
C18	0.20669 (15)	0.08174 (8)	−0.18286 (6)	0.0238 (2)	
H18A	0.0833	0.0859	−0.2010	0.029*	
H18B	0.2607	0.0385	−0.2179	0.029*	
C19	0.28879 (15)	0.17939 (8)	−0.18332 (7)	0.0248 (2)	
H19A	0.4026	0.1780	−0.1526	0.030*	
H19B	0.3040	0.1977	−0.2375	0.030*	
C20	0.25736 (17)	0.34043 (8)	−0.14647 (9)	0.0329 (3)	
H20A	0.3644	0.3391	−0.1117	0.049*	
H20B	0.1787	0.3855	−0.1265	0.049*	
H20C	0.2805	0.3597	−0.1988	0.049*	

### Atomic displacement parameters (Å^2^)

**Table:**

	*U*^11^	*U*^22^	*U*^33^	*U*^12^	*U*^13^	*U*^23^
O1	0.0249 (4)	0.0240 (4)	0.0367 (5)	0.0018 (3)	0.0058 (4)	0.0001 (3)
O2	0.0248 (4)	0.0227 (4)	0.0218 (4)	−0.0033 (3)	−0.0042 (3)	−0.0033 (3)
O3	0.0215 (4)	0.0161 (4)	0.0181 (4)	−0.0008 (3)	0.0002 (3)	−0.0014 (3)
O4	0.0308 (4)	0.0193 (4)	0.0175 (4)	−0.0038 (3)	0.0014 (3)	0.0012 (3)
O5	0.0235 (4)	0.0208 (4)	0.0497 (6)	0.0002 (3)	0.0120 (4)	0.0026 (4)
N1	0.0257 (5)	0.0205 (5)	0.0215 (5)	−0.0025 (4)	−0.0034 (4)	−0.0006 (3)
N2	0.0259 (5)	0.0198 (5)	0.0216 (5)	−0.0030 (4)	−0.0028 (4)	0.0002 (3)
C1	0.0361 (7)	0.0287 (6)	0.0355 (7)	0.0017 (5)	−0.0001 (6)	0.0022 (5)
C2	0.0234 (6)	0.0327 (6)	0.0227 (6)	0.0019 (5)	−0.0003 (4)	−0.0062 (4)
C3	0.0203 (5)	0.0274 (6)	0.0243 (6)	0.0003 (4)	−0.0035 (4)	−0.0091 (4)
C4	0.0174 (5)	0.0205 (5)	0.0248 (6)	0.0020 (4)	0.0043 (4)	−0.0037 (4)
C5	0.0204 (5)	0.0236 (6)	0.0314 (6)	−0.0018 (4)	0.0031 (5)	−0.0082 (4)
C6	0.0266 (6)	0.0173 (5)	0.0413 (7)	−0.0023 (4)	0.0079 (5)	−0.0040 (5)
C7	0.0300 (6)	0.0213 (6)	0.0335 (7)	0.0003 (4)	0.0070 (5)	0.0028 (4)
C8	0.0247 (6)	0.0208 (5)	0.0252 (6)	0.0008 (4)	0.0033 (4)	−0.0009 (4)
C9	0.0172 (5)	0.0186 (5)	0.0234 (6)	0.0012 (4)	0.0030 (4)	−0.0029 (4)
C10	0.0166 (5)	0.0197 (5)	0.0178 (5)	0.0022 (4)	−0.0001 (4)	−0.0025 (4)
C11	0.0169 (5)	0.0141 (5)	0.0218 (5)	0.0019 (4)	0.0025 (4)	0.0000 (4)
C12	0.0168 (5)	0.0156 (5)	0.0205 (5)	0.0032 (4)	0.0013 (4)	−0.0026 (4)
C13	0.0221 (5)	0.0191 (5)	0.0242 (6)	0.0020 (4)	0.0013 (4)	0.0016 (4)
C14	0.0238 (6)	0.0181 (5)	0.0328 (6)	−0.0025 (4)	0.0015 (5)	−0.0006 (4)
C15	0.0212 (5)	0.0218 (6)	0.0295 (6)	0.0004 (4)	−0.0022 (5)	−0.0075 (4)
C16	0.0221 (5)	0.0235 (5)	0.0199 (5)	0.0036 (4)	0.0010 (4)	−0.0039 (4)
C17	0.0180 (5)	0.0167 (5)	0.0212 (5)	0.0024 (4)	0.0038 (4)	−0.0015 (4)
C18	0.0278 (6)	0.0263 (6)	0.0170 (5)	0.0018 (4)	0.0008 (4)	0.0022 (4)
C19	0.0227 (6)	0.0262 (6)	0.0260 (6)	0.0028 (4)	0.0051 (5)	0.0057 (4)
C20	0.0271 (6)	0.0227 (6)	0.0492 (8)	−0.0015 (5)	0.0058 (6)	0.0042 (5)

### Geometric parameters (Å, °)

**Table:**

O1—C1	1.4149 (15)	C6—H6	0.9300
O1—C2	1.4171 (14)	C7—C8	1.3868 (16)
O2—C4	1.3577 (13)	C7—H7	0.9300
O2—C3	1.4360 (13)	C8—C9	1.3969 (15)
O3—C11	1.3593 (12)	C8—H8	0.9300
O3—C10	1.3663 (13)	C9—C10	1.4641 (15)
O4—C17	1.3594 (13)	C11—C12	1.4611 (14)
O4—C18	1.4341 (13)	C12—C13	1.3940 (15)
O5—C20	1.4154 (14)	C12—C17	1.4085 (15)
O5—C19	1.4158 (14)	C13—C14	1.3833 (15)
N1—C10	1.2996 (14)	C13—H13	0.9300
N1—N2	1.4057 (13)	C14—C15	1.3837 (17)
N2—C11	1.2967 (14)	C14—H14	0.9300
C1—H1A	0.9600	C15—C16	1.3845 (16)
C1—H1B	0.9600	C15—H15	0.9300
C1—H1C	0.9600	C16—C17	1.3952 (15)
C2—C3	1.4963 (17)	C16—H16	0.9300
C2—H2A	0.9700	C18—C19	1.4973 (16)
C2—H2B	0.9700	C18—H18A	0.9700
C3—H3A	0.9700	C18—H18B	0.9700
C3—H3B	0.9700	C19—H19A	0.9700
C4—C5	1.3913 (15)	C19—H19B	0.9700
C4—C9	1.4108 (15)	C20—H20A	0.9600
C5—C6	1.3870 (17)	C20—H20B	0.9600
C5—H5	0.9300	C20—H20C	0.9600
C6—C7	1.3882 (18)		
			
C1—O1—C2	112.44 (10)	N1—C10—O3	112.30 (9)
C4—O2—C3	117.88 (9)	N1—C10—C9	131.57 (10)
C11—O3—C10	102.96 (8)	O3—C10—C9	116.00 (9)
C17—O4—C18	117.63 (8)	N2—C11—O3	112.21 (9)
C20—O5—C19	111.58 (9)	N2—C11—C12	126.29 (9)
C10—N1—N2	105.90 (9)	O3—C11—C12	121.50 (9)
C11—N2—N1	106.62 (9)	C13—C12—C17	118.81 (10)
O1—C1—H1A	109.5	C13—C12—C11	117.27 (10)
O1—C1—H1B	109.5	C17—C12—C11	123.89 (9)
H1A—C1—H1B	109.5	C14—C13—C12	121.22 (11)
O1—C1—H1C	109.5	C14—C13—H13	119.4
H1A—C1—H1C	109.5	C12—C13—H13	119.4
H1B—C1—H1C	109.5	C13—C14—C15	119.41 (10)
O1—C2—C3	109.09 (10)	C13—C14—H14	120.3
O1—C2—H2A	109.9	C15—C14—H14	120.3
C3—C2—H2A	109.9	C14—C15—C16	120.79 (10)
O1—C2—H2B	109.9	C14—C15—H15	119.6
C3—C2—H2B	109.9	C16—C15—H15	119.6
H2A—C2—H2B	108.3	C15—C16—C17	120.01 (10)
O2—C3—C2	107.21 (9)	C15—C16—H16	120.0
O2—C3—H3A	110.3	C17—C16—H16	120.0
C2—C3—H3A	110.3	O4—C17—C16	123.53 (10)
O2—C3—H3B	110.3	O4—C17—C12	116.78 (9)
C2—C3—H3B	110.3	C16—C17—C12	119.69 (10)
H3A—C3—H3B	108.5	O4—C18—C19	107.28 (9)
O2—C4—C5	123.78 (10)	O4—C18—H18A	110.3
O2—C4—C9	116.32 (9)	C19—C18—H18A	110.3
C5—C4—C9	119.90 (10)	O4—C18—H18B	110.3
C6—C5—C4	119.94 (11)	C19—C18—H18B	110.3
C6—C5—H5	120.0	H18A—C18—H18B	108.5
C4—C5—H5	120.0	O5—C19—C18	109.63 (9)
C5—C6—C7	120.79 (11)	O5—C19—H19A	109.7
C5—C6—H6	119.6	C18—C19—H19A	109.7
C7—C6—H6	119.6	O5—C19—H19B	109.7
C8—C7—C6	119.43 (11)	C18—C19—H19B	109.7
C8—C7—H7	120.3	H19A—C19—H19B	108.2
C6—C7—H7	120.3	O5—C20—H20A	109.5
C7—C8—C9	120.94 (11)	O5—C20—H20B	109.5
C7—C8—H8	119.5	H20A—C20—H20B	109.5
C9—C8—H8	119.5	O5—C20—H20C	109.5
C8—C9—C4	118.89 (10)	H20A—C20—H20C	109.5
C8—C9—C10	118.81 (10)	H20B—C20—H20C	109.5
C4—C9—C10	122.26 (10)		
			
C10—N1—N2—C11	−0.40 (12)	N1—N2—C11—O3	1.21 (12)
C1—O1—C2—C3	−170.86 (10)	N1—N2—C11—C12	−177.78 (9)
C4—O2—C3—C2	−176.18 (9)	C10—O3—C11—N2	−1.48 (11)
O1—C2—C3—O2	76.04 (11)	C10—O3—C11—C12	177.56 (9)
C3—O2—C4—C5	−2.69 (15)	N2—C11—C12—C13	4.11 (16)
C3—O2—C4—C9	177.96 (9)	O3—C11—C12—C13	−174.79 (9)
O2—C4—C5—C6	178.67 (10)	N2—C11—C12—C17	−177.65 (10)
C9—C4—C5—C6	−2.00 (17)	O3—C11—C12—C17	3.44 (16)
C4—C5—C6—C7	−1.03 (18)	C17—C12—C13—C14	2.55 (16)
C5—C6—C7—C8	2.46 (18)	C11—C12—C13—C14	−179.12 (10)
C6—C7—C8—C9	−0.83 (18)	C12—C13—C14—C15	−0.30 (17)
C7—C8—C9—C4	−2.15 (17)	C13—C14—C15—C16	−1.66 (17)
C7—C8—C9—C10	175.47 (10)	C14—C15—C16—C17	1.30 (17)
O2—C4—C9—C8	−177.06 (9)	C18—O4—C17—C16	1.85 (15)
C5—C4—C9—C8	3.56 (16)	C18—O4—C17—C12	−177.53 (9)
O2—C4—C9—C10	5.40 (15)	C15—C16—C17—O4	−178.37 (10)
C5—C4—C9—C10	−173.98 (10)	C15—C16—C17—C12	0.99 (16)
N2—N1—C10—O3	−0.54 (12)	C13—C12—C17—O4	176.54 (9)
N2—N1—C10—C9	174.94 (10)	C11—C12—C17—O4	−1.68 (15)
C11—O3—C10—N1	1.22 (11)	C13—C12—C17—C16	−2.87 (15)
C11—O3—C10—C9	−175.03 (9)	C11—C12—C17—C16	178.92 (9)
C8—C9—C10—N1	−165.11 (11)	C17—O4—C18—C19	175.03 (9)
C4—C9—C10—N1	12.43 (18)	C20—O5—C19—C18	177.69 (10)
C8—C9—C10—O3	10.25 (14)	O4—C18—C19—O5	−75.52 (11)
C4—C9—C10—O3	−172.21 (9)		

### Hydrogen-bond geometry (Å, °)

**Table:**

*D*—H···*A*	*D*—H	H···*A*	*D*···*A*	*D*—H···*A*
C5—H5···N2^i^	0.93	2.62	3.385 (2)	140

Symmetry codes: (i) −*x*+1, *y*+1/2, −*z*+1/2.
